# Single blinded semi-field evaluation of MAÏA^®^ topical repellent ointment compared to unformulated 20% DEET against *Anopheles gambiae*, *Anopheles arabiensis* and *Aedes aegypti* in Tanzania

**DOI:** 10.1186/s12936-020-03461-9

**Published:** 2021-01-06

**Authors:** Emmanuel Mbuba, Olukayode G. Odufuwa, Frank C. Tenywa, Rose Philipo, Mgeni M. Tambwe, Johnson K. Swai, Jason D. Moore, Sarah J. Moore

**Affiliations:** 1 Vector Control Product Testing Unit, Department of Environmental Health and Ecological Science, Ifakara Health Institute, P.O. Box 74, Bagamoyo, Tanzania; 2 Swiss Tropical & Public Health Institute, Socinstrasse, 57, 4002 Basel, Switzerland; 3 University of Basel, St. Petersplatz 1, 4002 Basel, Switzerland; 4 London School of Hygiene and Tropical Medicine, Keppel St, London, WC1E 7HT UK

**Keywords:** Malaria, Mosquito, Topical repellent, Ointment, Protective efficacy, Complete protection time, CPT, DEET

## Abstract

**Background:**

*N*,*N*-Diethyl-3-methylbenzamide (DEET) topical mosquito repellents are effective personal protection tools. However, DEET-based repellents tend to have low consumer acceptability because they are cosmetically unappealing. More attractive formulations are needed to encourage regular user compliance. This study evaluated the protective efficacy and protection duration of a new topical repellent ointment containing 15% DEET, MAÏA^®^ compared to 20% DEET in ethanol using malaria and dengue mosquito vectors in Bagamoyo Tanzania.

**Methods:**

Fully balanced 3 × 3 Latin square design studies were conducted in large semi-field chambers using laboratory strains of *Anopheles gambiae *sensu stricto*, Anopheles arabiensis* and *Aedes aegypti.* Human volunteers applied either MAÏA^®^ ointment, 20% DEET or ethanol to their lower limbs 6 h before the start of tests. Approximately 100 mosquitoes per strain per replicate were released inside each chamber, with 25 mosquitoes released at regular intervals during the collection period to maintain adequate biting pressure throughout the test. Volunteers recaptured mosquitoes landing on their lower limbs for 6 h over a period of 6 to 12-h post-application of repellents. Data analysis was conducted using mixed-effects logistic regression.

**Results:**

The protective efficacy of MAÏA^®^ and 20% DEET was not statistically different for each of the mosquito strains: 95.9% vs. 97.4% against *An. gambiae* (OR = 1.53 [95% CI 0.93–2.51] p = 0.091); 96.8% vs 97.2% against *An. arabiensis* (OR = 1.08 [95% CI 0.66–1.77] p = 0.757); 93.1% vs 94.6% against *Ae. aegypti* (OR = 0.76 [95% CI 0.20–2.80] p = 0.675). Average complete protection time (CPT) in minutes of MAÏA^®^ and that of DEET was similar for each of the mosquito strains: 571.6 min (95% CI 558.3–584.8) vs 575.0 min (95% CI 562.1–587.9) against *An. gambiae*; 585.6 min (95% CI 571.4–599.8) vs 580.9 min (95% CI 571.1–590.7) against *An. arabiensis*; 444.1 min (95% CI 401.8–486.5) vs 436.9 min (95% CI 405.2–468.5) against *Ae. aegypti.*

**Conclusions:**

MAÏA^®^ repellent ointment provides complete protection for 9 h against both *An. gambiae* and *An. arabiensis*, and 7 h against *Ae. aegypti* similar to 20% DEET (in ethanol). MAÏA^®^ repellent ointment can be recommended as a tool for prevention against outdoor biting mosquitoes in tropical locations where the majority of the people spend an ample time outdoor before going to bed.

## Background

The use of insecticide-treated bed nets (ITNs) and indoor residual sprays (IRS) has almost halved malaria burden throughout sub-Saharan Africa [1, 2]. However, a considerable proportion of malaria transmission occurs outside of sleeping hours when ITNs are not in use [3]. Topical mosquito repellents are one of the oldest interventions used to prevent contact between humans and mosquito vectors [4, 5], and may be useful for people who spend their time outdoors in the evening or overnight for occupational or social activities [6–9]. Some studies have shown that people who use topical repellents consistently in addition to ITNs are protected from malaria [10, 11]. However, their utility as a disease prevention tool is limited by poor user compliance [12]. Therefore, deployment of topical mosquito repellents for malaria prevention is not recommended by the World Health Organization (WHO) as an intervention with public health value but maybe beneficial as an intervention to provide additional personal protection against malaria [13, 14].

Topical repellents must be applied daily or even several times a day, and poor user compliance is a major limitation to the effectiveness of topical repellents [12, 15, 16]. Common reasons for poor compliance include forgetting to apply the repellent [15, 17], poor acceptability including unpleasant smell or greasy “feel” on the skin [17] and the perception that repellents are poisonous [15]. DEET is a common active ingredient of most of the topical mosquito repellents present in the market today. Over 200 million persons use DEET-based repellents every year with the market growing annually due to increases in vector-borne diseases, such as dengue and Zika as well as nuisance of the vector [18]. DEET has an excellent safety profile and is safe for use among children and pregnant women [19, 20].

According to Carroll et al. [21] “a good repellent ointment should be effective against target vector strain, easy to apply and has a nice odour and the residual feeling after application”. To obtain better compliance, consideration of human customs and behaviour that may encourage consistent use of repellents is essential. In a study conducted in Burkina Faso, it was observed that about 91% of children under 5-years were washed in the evening and 80% of them receive ointment on their skin after bathing and before bed time (Traoré et al.; pers. commun*.*). During this time, mosquitoes are actively host-seeking and interacting with humans outdoors [22–24]. Therefore, a well-formulated topical mosquito repellent with skin softening properties for daily use after bathing may improve user compliance as well as protect against vector-borne diseases, such as dengue and malaria, when people are outdoors in the early evening.

The Maïa Africa SAS [25], a company based in Burkina Faso worked in collaboration with local mothers to develop MAÏA^®^ repellent ointment that is formulated with petroleum jelly, shea butter, cotton oil, beeswax, fragrance, and 15% DEET. Shea butter-based ointments are widely used for skin softening purposes [26, 27] and MAÏA^®^ repellent is designed to repel mosquitoes in addition to softening the human skin. This study evaluated the protective efficacy and duration of repellency of MAÏA^®^ repellent ointment in comparion to the gold standard, unformulated 20% DEET against two species of *Anopheles*, i.e. *Anopheles gambiae* and *Anopheles arabiensis*, and *Aedes aegypti* under semi-field system conditions in Bagamoyo Tanzania. The semi-field evaluation of topical repellents generates data that are comparable to a full-field evaluation [28].

## Methods

### Study area

**Fig. 1:**
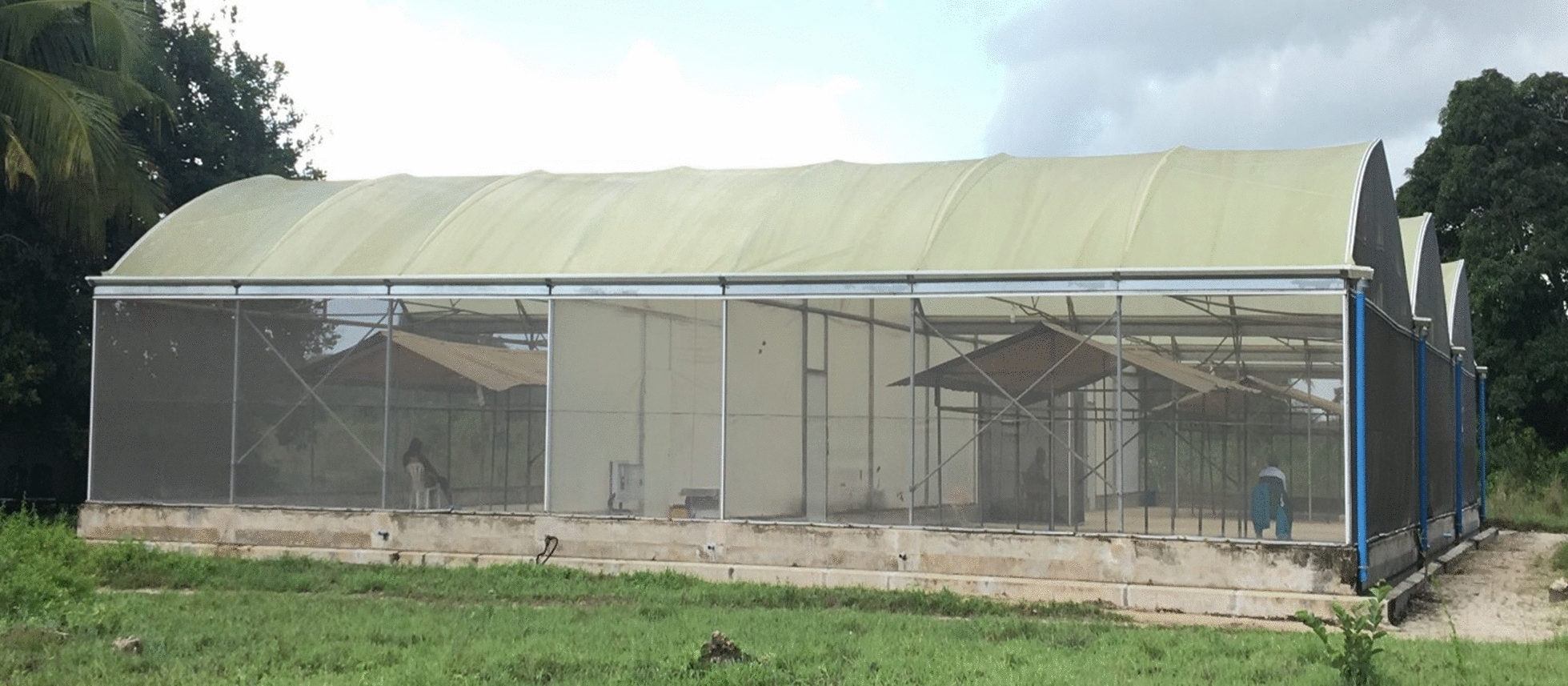
A semi-field system (SFS). A semi-field system (SFS) with 29 × 21 m built from a fabricated greenhouse frame modified to make two chambers with a central corridor and an opaque polyethylene roof for rain protection

This study was conducted under ambient conditions in a semi-field system measuring 29 × 21 m built from a fabricated greenhouse frame modified to make two compartments with a central corridor and an opaque polyethylene roof for rain protection (Fig. 1) [28]. The semi-field system (SFS) is located at 6°8′ S, 30°37′ E at the Ifakara Health Institute in Bagamoyo district in Tanzania. Bagamoyo district experiences annual rainfall between 800 and 1000 mm, temperatures between 22 and 33 °C, and mean relative humidity of 73%. This evaluation followed the WHO Guidelines for efficacy testing of topical repellents [29].

### Study design

This study was divided into two parts to accommodate the circadian rhythm of mosquitoes investigated. *An. gambiae *sensu stricto (s.s.) and *An. arabiensis* were tested at night and *Ae. aegypti* in the early morning*.* A pilot study for the first 6 h (h) after repellent application was conducted and found that a longer testing period was required. The final study tested the repellent from 6 to 12 h post-application.

### A study for *Anopheles*

**Fig. 2:**
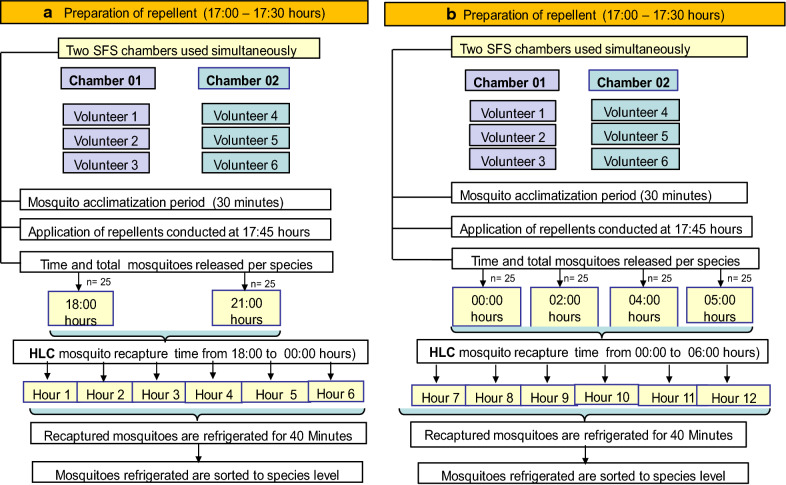
Study flow for *Anopheles*. Study flow for *An. gambiae* s.s. and *An. arabiensis* conducted for 6 h and 12-h of recapture period. Two semi-field system (SFS) chambers (1 and 2) were used and two fully balanced Latin square (LS) design (3 × 3) were conducted simultaneously using six human volunteers. Mosquito recapture started immediately after application of repellent in the pilot study (**a**) and started 6-h post-application of repellents in the final study (**b**). Total mosquitoes of each strains released were 50 (25 per release) and 100 (25 per release) in the pilot and final study, respectively

In the pilot study of *Anopheles* strains, repellents were applied at 17:45 h and mosquito collection was conducted between 18:00 and 00:00 h (Fig. 2a). In the final study, repellents were applied at 17:45 h and mosquito collection was conducted between 00:00 and 06:00 h (i.e. 6–12 h after the application of repellents) (Fig. 2b).

Both the pilot and final study consisted of two fully balanced (3 × 3) Latin squares (LS) design conducted in two chambers of the SFS simultaneously over nine nights using six volunteers. In each LS, three volunteers rotated sequentially between the three mosquito collections positions each day inside the chamber and swapped repellents after every 3-days. After 9 days of the study period, each volunteer had tested each of the repellents at each of the three mosquito collection positions inside the SFS chamber three times. The study flow plan for *An. gambiae* s.s. and *An. arabiensis* is shown in (Fig. 2).

### A study for Aedes

**Fig. 3:**
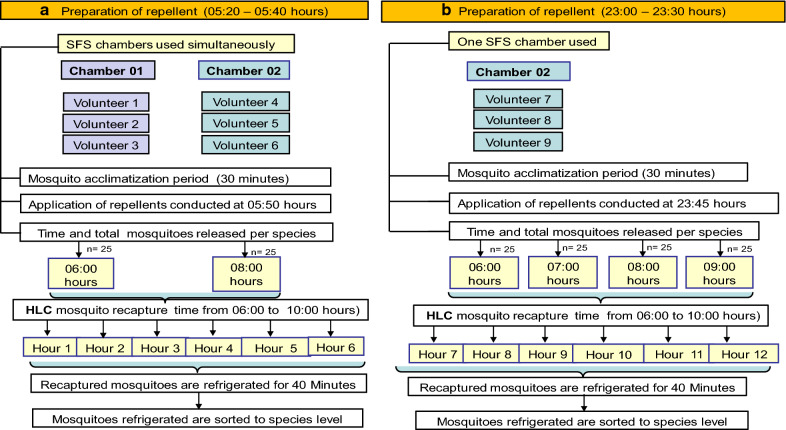
Study flow for *Aedes aegypti*. Study flow plan for *Ae. aegypti* experiment conducted for 6 h and 12 h of recapture period. Two semi-field system (SFS) chambers (1 and 2) were used simultaneously and two fully balanced Latin square (LS) design (3 × 3) were conducted using six human volunteers in a pilot study. Mosquito recapture started immediately after application of repellent in the pilot study (**a**) and started 6 h post-application of repellents in the final study (**b**). Total female *Ae. aegypti* released were 50 (25 per release) and 100 (25 per release) in the pilot and final study, respectively

For *Ae. aegypti*, repellents were applied at 05:50 h in the pilot study and at 23:45 h in the final study. Mosquito collection was conducted from 06:00 to 10:00 h in both the pilot and final study. The pilot study consisted of two fully balanced (3 × 3) LS conducted in two chambers of the semi-field system simultaneously over nine nights using six volunteers. In the final study, one fully balanced (3 × 3) LS was conducted in one chamber over nine nights using three additional volunteers. In both studies, volunteers rotated sequentially between three collection positions each day inside the chamber of the SFS and switched repellents after every 3-days. After the 9-days study, each volunteer had tested each of the repellents at each of the three mosquito collection positions inside the SFS chamber three times. The study flow plan for *Ae. aegypti* is shown in Fig. 3.

### Mosquito strains

This study used nulliparous female laboratory-reared mosquitoes, aged 5–7 days old, sugar starved and reactive to human odour. Mosquito strains used were pyrethroid-resistant (16% mortality to 0.05 deltamethrin, 15% mortality to 0.05 alphacypermethrin and 9% mortality to 0.75 permethrin) *An. arabiensis* (Kingani, colonized Tanzania 2006), fully pyrethroid susceptible *An. gambiae* (Kisumu, colonized Kenya 1975) and fully pyrethroid susceptible *Ae. aegypti* (Bagamoyo, colonized Tanzania 2018). Mosquitoes were reared following the MR4 guideline [30]. Before the experiment, *An. arabiensis* were lightly marked by placing them in a cup coated with a fluorescent dye to make them distinguishable from the morphologically identical *An. gambiae*. By very lightly marking the mosquitoes there was no significant effect on their fitness nor host preference [31]. Mosquitoes were then sugar-starved for 8 h. About 30 min before the experiment, 100 female mosquitoes that were responsive to human odour were selected and transported in boxes to the SFS chambers to acclimatize with the ambient environmental conditions.

### Repellents tested

MAÏA^®^ ointment and 20% DEET were shipped to Ifakara Health Institute (IHI) Vector Control Product Testing Unit (VCPTU) in plastic jars by MAÏA Africa SAS [25]. The amount of MAÏA^®^ repellent ointment and DEET 97% (reference number 26028, lot number 2436308) received at IHI was 600 and 200 ml respectively. After the products were received, they were stored the same day at room temperature between 25 and 29 °C until used in the experiment. The 20% DEET in ethanol (V/V) was prepared in-house before the experiment.

### Volunteers

Nine male volunteers between the age of 24 and 30 years were recruited after signing informed consent forms written in Kiswahili. All volunteers were tested for malaria parasite infection using SD BIOLINE Malaria Ag P.f [32] rapid malaria diagnostic kits before participating in the study and once per week during the study period as part of IHI health and safety procedures. Only male volunteers were recruited for cultural reasons.

### Allocation of volunteers

**Fig. 4:**
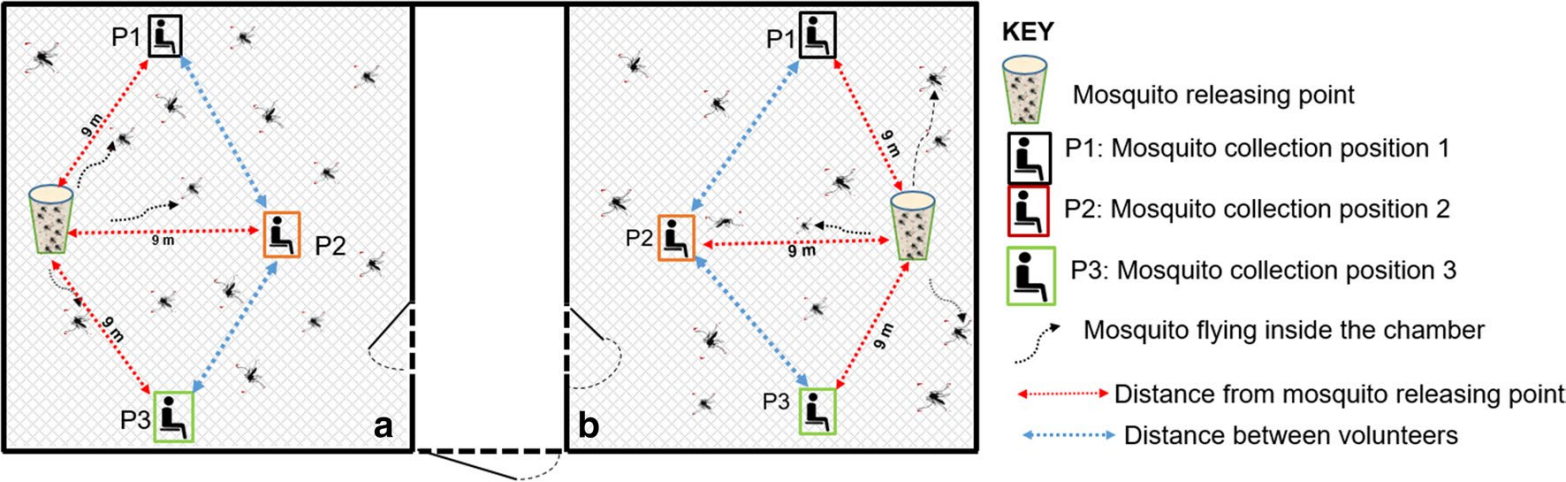
An experimental set up in a semi-field system. A schematic diagram of a semi-field system showing two chambers (**a**, **b**), three mosquito collection positions and one mosquito releasing point inside each chamber. The distance between releasing point and mosquito collection positions was nine meters

At the beginning of the study (pilot study), six male volunteers were assigned into two groups of 3 volunteers. One group was assigned to chamber ‘1’ and the other in chamber ‘2’ of the SFS (Fig. 1). Inside each chamber, three mosquito recapture positions were marked each 9 m from the mosquito releasing point (Fig. 4). Each volunteer was randomly assigned to one of the three mosquito recapture positions inside one chamber and rotated between positions nightly. After 9 days of experiments, it was discovered that the complete protection time of both repellents was above 6 h. Therefore, the final study of 12 h recapture period was set up with an additional three volunteers recruited to test the investigational product against *Ae. aegypti*, which was conducted immediately after the *Anopheles* experiment.

### Application of repellents

Volunteers wore shorts and washed their lower limbs using water without soap before starting the experiment and they also wore closed shoes and a mesh bug jacket to ensure mosquitoes have access to the lower limbs only. Volunteers were non-smokers, and were requested to not drink alcohol or use perfumed soaps or ointment during the entire study period. We calculated a lower limb-skin surface area for each volunteer using the following formula at the beginning of the study; Area = ½ (K + A) × L.

Where “L” represents the leg length between the knee and the ankle, “K” represents the circumference at the knee and “A” represents the circumference of the ankle area. The average lower limb skin-surface area of volunteers was 1259.2 cm^2^. The amount of repellents used on the day of experiment was measured using Ohaus CS200 weighing scale (Ohaus Corporation, USA), in which plastic cups that contained MAÏA^®^ were weighed on the scale before and after application to determine the amount of repellents used. The average amount of MAÏA^®^ repellent ointment applied per limb was 2.52 g corresponding to a target dose of 2 mg/cm^2^ (approximately 0.3 mg of DEET per cm^2^). The average amount of 20% DEET in ethanol applied per limb was 2.4 g corresponding to 1.9 mg/cm^2^ (approximately 0.38 mg of DEET per cm^2^). All volunteers applied repellents using latex-gloved hands to minimize absorption onto the hands. Repellents for the *Anopheles* study were applied at 17:45 h and for the *Aedes* study at 05:50 h during the pilot study, while during the final study repellents were applied at 17:45 h for the *Anopheles* study and at 23:45 h for the *Aedes* study. In the final study, all participants rested with their trousers rolled up to prevent abrasion of the repellents after repellent application.

### Duration of the study

#### Six-hour pilot test

During the first 9 days, 50 *An. arabiensis* and 50 *An. gambiae* were released in each of the SFS chambers and testing was conducted for 6 h. During the 6 h recapture period, no mosquitoes were recaptured by volunteers who applied MAÏA^®^ and 20% DEET. Therefore, the recapture period was extended to 12-h in order to confidently determine the duration of complete protection time of MAÏA^®^ repellent ointment and 20% DEET.

#### Twelve-hour test

During the LS with 12-h of recapture period, the same volunteers, chambers and rotation schedule was used for *Anopheles* experiment: 100 *An. arabiensis* and 100 *An. gambiae* were released (25 per release) starting at 00:00 h. Three additional volunteers were recruited following study procedure and were assigned to the *Ae. aegypti* experiments in which 100 *Ae. aegypti* were released with 25 released every hour between 06:00 and 10:00 h.

### Mosquito recapture

Volunteers recorded the time of a first mosquito recapture of each experiment (*Anopheles* or *Aedes*) and placed in a separate cup labelled with the time of recapture, volunteer’s code, position and repellent (treatment) code. Volunteers collected subsequent mosquitoes that landed for a 6-h collection period up to 12-h post-application of repellent with cups labelled with repellent code, position and hour of collection. Cups were changed after every hour. At the end of recapture time, mosquitoes were killed by refrigeration at − 4 °C for about 40 min and then sorted to species level. If fewer than 50% of mosquitoes were recaptured by a negative control volunteer, the data were discarded and the replicate was repeated. Temperature, relative humidity and wind speed were recorded on the day at the beginning of the experiment.

### Data management and statistical analysis

Data were recorded in paper forms and then double entered and cleaned in Microsoft Excel 2016. Data analyses were performed in Stata 15.1 (Stata Corp, USA). Descriptive analysis of mosquitoes recaptured by repellents was performed. The mean complete protection time (CPT) of each repellent for each mosquito strain was estimated using the Kaplan–Meier survival analysis. The protective efficacy (PE) was established for the data collected up to 12-h and calculated using this formula; P = ((C − T)/C) * 100; where ‘P’ represents the percentage protection, ‘C’ represents the number of mosquitoes recaptured on the negative control (ethanol) and ‘T’ represents the number of mosquitoes recaptured on volunteer’s lower limbs treated with either MAÏA^®^ or 20% DEET.

Statistical analysis was performed using a mixed-effects binary logistic regression to compare the protective efficacy between MAÏA^®^ and 20% DEET (as the reference in the statistical model). Several models were tested using the proportion of recaptured mosquitoes as the outcome variable and repellent type (treatments), volunteer, position of the volunteer and time of recapture as fixed effects and day of test as random effects. The best-fit model was determined using the Aikaike’s Information Criterion (AIC) and the model with the smallest AIC value was selected. In the *Anopheles* data, repellent and time after application were fixed effects and the day of test was a random effect. In the *Aedes* data, the type of repellent, time of recapture and position of the volunteer were fixed effects and the day of test was a random effect.

## Results

### General test conditions

The average environmental conditions for *Anopheles* experiment was 26.5 °C (95% CI 26.4–26.6) temperature, 82.96% (95% CI 82.4–83.5) relative humidity, and 0.36 m/s (95% CI 0.3–0.4) wind speed, and for *Ae. aegypti* experiment, temperature was 24.6 °C (95% CI 24.5–24.7), relative humidity 59.45% (95% CI 55.7–63.2), and wind speed 0.00 m/s (95% CI 0.0–0.0). All tests were conducted with recapture rate in the negative control arm exceeding 50%.

### Descriptive analysis

The percentage recapture for *An. gambiae* was 69.9% (1258/1800) and that of *An. arabiensis* was 75.4% (1357/1800) during the 12 h tests. The percentage recapture for *Ae. aegypti* was 88.9% (800/900) (Table 1). The geometric mean (GM) hourly mosquito landings are shown in Table 1. The GM hourly landings on volunteers who applied absolute ethanol was 9.46 (95% CI 8.4–10.6) for *An. gambiae*, 10.2 (95% CI 9.0–11.5) for *An. arabiensis*, and 10.0 (95% CI 8.3–12.1) for *Ae. aegypti.* There was no variability between volunteers in terms of mosquito attractiveness but most users preferred using MAÏA^®^ repellent ointment than 20% DEET (in ethanol).

**Table 1.** The percentage (%) recapture, geometric mean of hourly mosquito landings, percentage protection, mean complete protection time (CPT) and odds ratio between MAÏA^®^ repellent ointment, 20% DEET and absolute ethanol

Test systems (mosquito strain)	Test items (repellents)	Percentage recapture	Geometric mean hourly landings	Percentage protection (PE) and 95% CI	Odds ratio	P value	95% confidence interval	Mean CPT in minutes (95% CI)
Susceptible *An. gambiae *s.s.	20% DEET	2.5% (31/1258)	1.21 (1.05–1.40)	97.4% (97.1–97.6)	1	–	–	575.0 (562.1–587.9)
	MAÏA^®^ ointment	3.9% (49/1258)	1.57 (1.28–1.94)	95.9% (95.4–96.3)	1.53	0.091	0.93–2.51	571.6 (558.3–584.8)
	Absolute ethanol	93.6% (1178/1258)	9.46 (8.43–10.60)	–	62.63	0.00001	40.04–97.97	–
Resistant *An. arabiensis*	20% DEET	2.7% (36/1357)	1.40 (1.15–1.71)	97.2% (96.9–97.4)	1	–	–	580.9 (571.1–590.7)
	MAÏA^®^ ointment	3% (40/1357)	1.52 (1.18–1.96)	96.8% (96.3–97.3)	1.08	0.757	0.66–1.77	585.6 (571.4–599.8)
	Absolute ethanol	94.4% (1281/1357)	10.17 (9.01–11.48)	–	58.93	0.00001	38.72–89.71	–
*Ae. Aegypti*	20% DEET	4.8% (38/800)	3.36 (2.35–4.82)	94.6% (93.8–95.4)	1	–	–	436.9 (405.2–468.5)
	MAÏA^®^ ointment	6% (48/800)	2.47 (1.7–3.5)	93.1% (92.2–94.1)	0.76	0.675	0.20–2.80	444.1 (401.8–486.5)
	Absolute ethanol	89.3% (714/800)	10.0 (8.30–12.12)	–	14.87	0.00001	6.99–31.65	–

### The protective efficacy of MAÏA^®^ and 20% DEET

For the recapture period, 6–12 h post-application, both MAÏA^®^ and 20% DEET provided greater than 93% protective efficacy against all strains (Table 1). There was no significant difference in the protective efficacy (PE) between the MAÏA^®^ repellent ointment and the unformulated 20% DEET (in ethanol) over the 12-h test period for any of the mosquito strains tested. For *An. gambiae *s.s., MAÏA^®^ PE was 95.9% (95% CI 95.4–96.3) and 20% DEET's PE was 97.4% (95% CI 97.1–97.6), OR = 1.53 [95% CI 0.93–2.51] p = 0.091. For *An. arabiensis,* MAÏA^®^ PE was 96.8% (95% CI 96.3–97.3) and 20% DEET's PE was 97.2% (95% CI 96.9–97.4), OR = 1.08 [95% CI 0.57–2.04] p = 0.757. For *Ae. aegypti*, MAÏA^®^ PE was 93.1% (95% CI 92.2–94.1) and 20% DEET's PE was 94.6% (95% CI 93.8–95.4), OR = 0.76 [95% CI 0.20–2.80] p = 0.675 (Table 1).

### Complete protection time (minutes) of MAÏA^®^ and 20% DEET

**Fig. 5:**
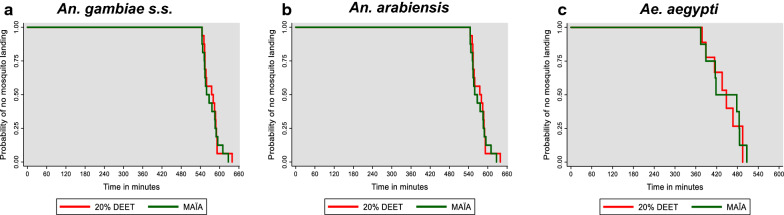
Complete protection time (CPT) of MAÏA^®^ repellent ointment and 20% DEET against laboratory-reared mosquito strains. **a** Probability of no *An. gambiae* landing on lower limbs of volunteers treated with MAÏA^®^ repellent ointment (green) and 20% DEET (red). **b** Probability of no *An. arabiensis* landing on lower limbs of volunteers treated with MAÏA^®^ repellent ointment (green) and 20% DEET (red). **c** The probability of no *Ae. aegypti* landing on lower limbs of volunteers treated with MAÏA^®^ repellent ointment (green) and 20% DEET (red)

MAÏA^®^ repellent ointment and unformulated 20% DEET had similar complete protection time (CPT) exceeding 9 h (Table 1, Fig. 5). The average CPT (minutes) of MAÏA^®^ was 571.6 (95% CI 558.3–584.8) and 20% DEET was 575.0 (95% CI 562.1–587.9) against *An. gambiae *s.s. Average CPT (minutes) of MAÏA^®^ was 585.6 (95% CI 571.4–599.8) and 20% DEET was 580.9 (95% CI 571.1–590.7) for *An. arabiensis*. Average CPT (minutes) was 444.1 (95% CI 401.8–486.5) and 20% DEET was 436.9 (95% CI 405.2–468.5) for *Ae. aegypti* (Fig. 5).

## Discussion

DEET-based topical mosquito repellents provide personal protection against numerous arthropods of medical importance including malaria vectors, such as *An. gambiae* and *An. arabiensis* and the dengue vector *Ae. aegypti* [33]. The evaluation of the protective efficacy and duration of protection of MAÏA^®^ repellent ointment compared to a standard unformulated 20% DEET in ethanol under semi-field conditions was done in the SFS. Sangoro et al*.* [28] demonstrated that a semi-field evaluation of topical mosquito repellent gives similar results to field studies but is far safer as only disease-free laboratory-reared mosquitoes are used. This is especially important in the case of *Ae. aegypti* evaluations in areas of active dengue transmission, such as Tanzania [34]. Moreover, semi-field environments allow volunteers to be accessible to a known number of mosquitoes of known age, physiological status, and avidity, and minimize heterogeneity in the data, allowing a more precise estimation of true repellent efficacy. The study is also conservative as it used young, never blood-fed mosquitoes raised under optimal conditions to maximize body size and then sugar starved, which are less repelled by DEET than older or smaller mosquitoes [35, 36].

The study results demonstrated that MAÏA^®^ repellent ointment is comparable to unformulated 20% DEET in terms of mean repellency over 12 h as well as the duration of CPT against both malaria and dengue vectors. MAÏA^®^ repellent ointment and 20% DEET had CPT of more than 9-h against *An. gambiae* and *An. arabiensis* and more than 7-h for *Ae. aegypti* post-application of the repellents. These results are similar to another study which evaluated the effectiveness of MAÏA^®^ repellent ointment under field conditions in Burkina Faso in which the authors concluded that MAÏA^®^ and 20% DEET are comparable in terms of duration of CPT against *Anopheles* strains and *Ae. aegypti* (Guelbeogo et al. pers. commun*.*).

The study results indicated that MAÏA^®^ repellent ointment is an effective mosquito repellent suitable for the use even under high mosquito biting pressure. According to Goodyer et al. [37] an ideal mosquito repellent should provide CPT greater than 6-h under the highest mosquito biting pressure. The present study fulfilled this characteristic by demonstrating the mean CPT above 9-h against *An. gambiae* and *An. arabiensis* and above 7-h against *Ae. aegypti* for both MAÏA^®^ and 20% DEET with an average of 10 mosquito landings per strain per hour in the control.

User compliance is a major limitation of most topical insect repellents [12, 15, 16] and since topical repellents are applied to the skin, most users prefer insect repellents which are cosmetically pleasant in terms of odour and feel on the skin; in addition to providing protection from biting insects [38]. In this study, all volunteers preferred to use MAÏA^®^ repellent ointment compared to 20% DEET because those who applied MAÏA^®^ repellent reported that the ointment felt better on their skin. This observation confirms that some repellent users may be influenced by product characteristics such as texture, skin feel, and odour [38, 39]. DEET tends to damage some plastics [40] which is a disadvantage in an area where plastic footwear is commonly worn. An additional benefit observed by the users was that MAÏA^®^ repellent ointment did not damage plastics when intentionally applied to a plastic watch strap. Therefore, the use of MAÏA^®^ repellent ointment may be a suitable alternative to a less cosmetically appealing DEET-based formulations. However, more studies are required to specifically assess user acceptability of MAÏA^®^ repellent ointment compared to other formulated products available on the market and whether this may improve compliance.

## Study limitations

After the initial 9 days of the experiment, the study was extended for another 9 days to confidently determine the CPT of MAÏA^®^ repellent ointment and 20% DEET. This led to the addition of volunteers to work exclusively on the *Aedes* experiment conducted in one chamber of the SFS and only one Latin square design conducted. Therefore, the mean CPT of MAÏA^®^ repellent ointment and 20% DEET was achieved using different and fewer (3) volunteers in the *Aedes* experiment compared to six volunteers and two SFS chambers used simultaneously in the *Anopheles* experiments. Ideally, the experiment would use more volunteers to capture the repellent efficacy against a wide range of people [41]. The study did not asses the efficacy of the repellent against nuisance mosquitoes, such as *Culex quinquefasciatus*, which may also be important when assessing the consumer acceptance of repellents. This study did not involve female participants although DEET is generally more protective against women than men due to their smaller size that makes them less attractive to mosquitoes [42].

## Conclusion

In conclusion, MAÏA^®^ repellent ointment is comparable to unformulated 20% DEET under high biting pressure. Therefore, it may be recommended for use in disease-endemic areas as it protects for more than 6 h. It is a cosmetically appealing mosquito bite protection tool that also nourishes and moisturizes the skin, which may improve consumer acceptability and fit into daily life if used every evening after bathing.

## Data Availability

Data are available upon reasonable request from the corresponding author.

## Abbreviations

AIC: Aikaike’s Information Criterion
CI: Confidence interval
CPT: Complete protection time
DEET: *N*,*N*-Diéthyl-3-methylbenzamide
GM: Geometric mean
HQ: Headquarter
IHI: Ifakara Health Institute
IRB: Institutional Review Board
LS: Latin square
NIMR: National Institute for Medical Research
PE: Protective efficacy
SFS: Semi field system
V/V: Volume/volume
VCPTU: Vector Control Product Testing Unit
WHO: World Health Organization
